# Intraocular pressure after combined photorefractive keratectomy and corneal collagen cross-linking for keratoconus

**DOI:** 10.1007/s10792-023-02886-w

**Published:** 2023-10-20

**Authors:** Karim Mahmoud Nabil, Ahmed Abdel Karim Elmassry, Silas Ntakarusho, Amr Ahmed Said

**Affiliations:** 1 Department of Ophthalmology, Faculty of Medicine, University of Alexandria, Postal Code 21523 19 Amin Fekry Street, Raml Station Alexandria, Egypt; 2 Department of Ophthalmology, University of Ngozi, Ngozi, Burundi

**Keywords:** Keratoconus, Photorefractive keratectomy, Corneal collagen cross linking, Intraocular pressure

## Abstract

**Purpose:**

The purpose of this prospective study was to evaluate the effect of combined photorefractive keratectomy (PRK) and corneal collagen cross-linking (CXL) on intraocular pressure (IOP) in patients with keratoconus (KC).

**Methods:**

We included 64 eyes of 34 patients (19 males and 15 females; age: 19-40y) with stages 1–2 keratoconus which had undergone combined wavefront-optimized photorefractive keratectomy and corneal collagen cross linking. Two other groups of patients were added as controls: the PRK group including 110 eyes of 57 patients (23 males and 34 females; age: 18-44y) which had undergone wavefront-optimized photorefractive keratectomy for myopic refractive errors, and the CXL group including 36 eyes of 23 patients (14 males and 9 females; age: 12-38y) with keratoconus, not filling the inclusion criteria for combined PRK and CXL, which had undergone corneal collagen cross-linking. IOP was recorded preoperatively and postoperatively at 3, 6 and 12 months follow-up visits.

**Results:**

Preoperative IOP in both CXL (12.1 ± 2.53 mmHg) and PRK + CXL (13.2 ± 2.50 mmHg) groups was significantly lower than PRK group (15.8 ± 3.10 mmHg) (F = 30.505, *p* < 0.001). At 3 months postoperatively, IOP showed no statistically significant difference between the three studied groups (F = 1.821, *p* = 0.164). At 6 months postoperatively, IOP in the CXL group (14.6 ± 2.64 mmHg) was significantly higher than both PRK (13.4 ± 2.27 mmHg) and PRK + CXL (13.3 ± 2.62 mmHg) groups (F = 3.721, *p* = 0.026). At 12 months postoperatively, IOP in the CXL group (14.3 ± 2.69 mmHg) was significantly higher than the PRK group (13.2 ± 2.23 mmHg) and was higher than PRK + CXL group (13.3 ± 2.59 mmHg) although not statistically significant (F = 3.393, *p* = 0.035).

Regarding the percent of change from preoperative IOP, a statistically significant difference between the three studied groups was detected at 3, 6 and 12 months postoperatively (H = 117.459, 109.303, 122.694 respectively, *p* < 0.001). The median percent of change from preoperative IOP in the PRK group was −16.7%, −15%, and −16.7%, in the CXL group was + 14.3%, + 19.4%, and + 19.1%, while in PRK + CXL group was 0% at 3, 6 and 12 months postoperatively. (Post-hoc power analysis 75%).

**Conclusions:**

Combined PRK and CXL in patients with KC shows no significant effect on IOP, in contrast to either procedure performed separately.

## Introduction

Keratoconus is a bilateral progressive, usually asymmetric, non-inflammatory corneal ectatic disorder, presenting predominantly in adolescence, characterized by apical corneal bulging, central corneal thinning, and corneal distortion [1, 2]. With disease progression, irregular astigmatism and ocular aberrations increase, reducing image quality and visual acuity, and may be complicated by apical corneal scarring in advanced cases [1, 2].

The main aims of keratoconus treatment involve the arrest of the progression of corneal ectasia, refractive error reduction, and restoration of the corneal shape to normal [3, 4]. A promising therapeutic option is corneal collagen cross-linking (CXL), which uses ultraviolet A (UVA) to activate riboflavin, creating covalent bonds between collagen fibrils, increasing biomechanical corneal stiffness and restoring its tensile strength [3]. A combined treatment approach, consisting of photorefractive keratectomy (PRK) with CXL, should have superior efficacy in keratoconus management, since this combination involves both corneal strengthening and halting keratoconus progression by CXL and reducing the refractive error by PRK [1].

Multiple studies concluded that CXL increased corneal stiffness, resulting in an overestimation of intraocular pressure (IOP) [5, 6]. On the other hand, IOP showed significant reduction after myopic PRK, strongly linked to the depth of tissue ablation and the dioptric power of myopia correction [7].

In this study, we evaluated the effect of combined photorefractive keratectomy and corneal collagen crosslinking on intraocular pressure in patients with keratoconus.

### Patients and methods

All patients were recruited from the Department of Ophthalmology, Faculty of Medicine, Alexandria University (Alexandria, Egypt). Informed consent was obtained from all patients. This study was approved by the Ethics of Research Committee, Faculty of Medicine, University of Alexandria, Egypt.

**Fig. 1:**
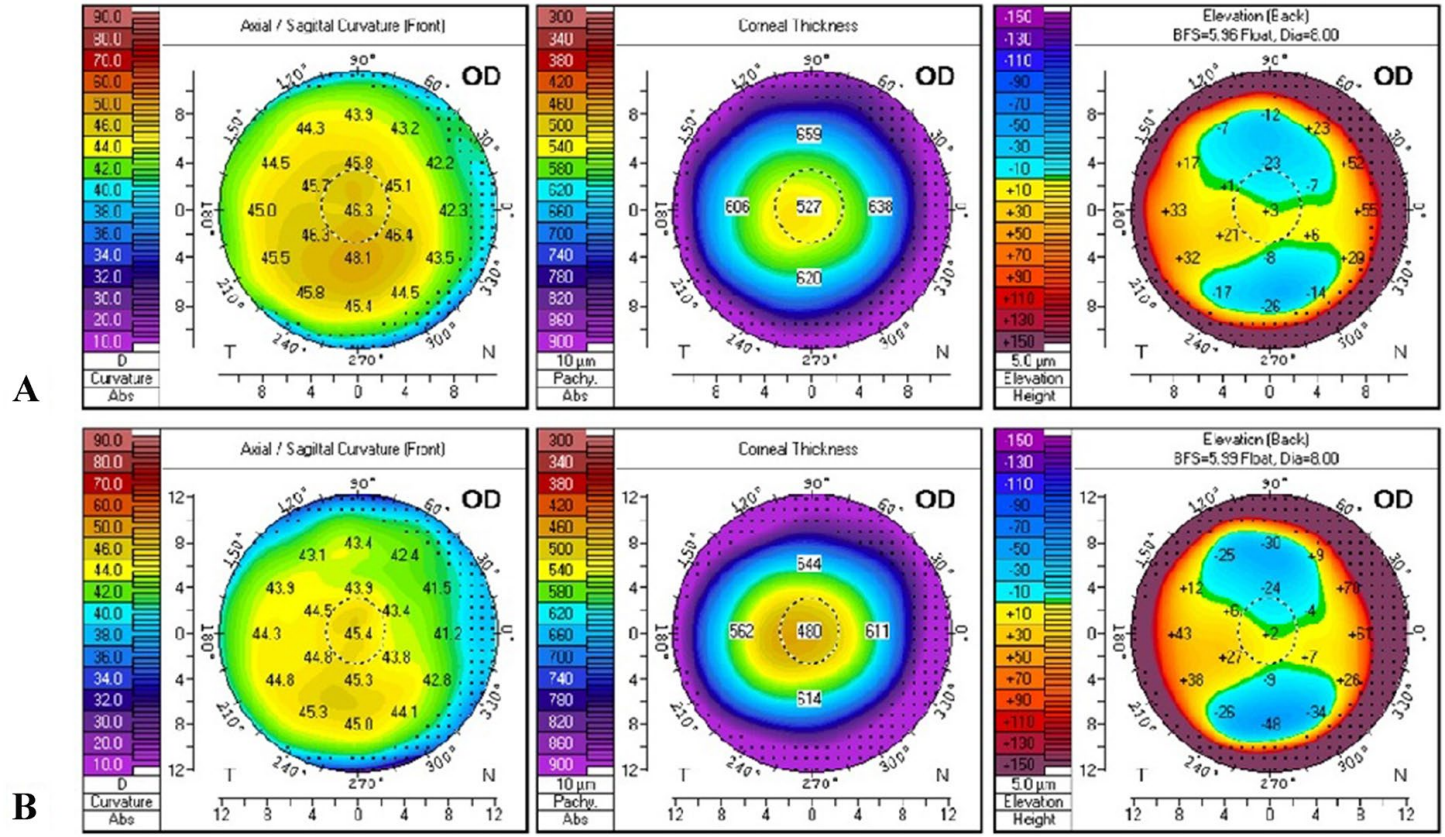
**A**: Preoperative, **B**: 6 months post PRK + CXL Scheimpflug tomography of stage 1 KC patient (correction -0.75 D sphere, -0.75 D cylinder, ablation depth 24 µ)

**Fig. 2:**
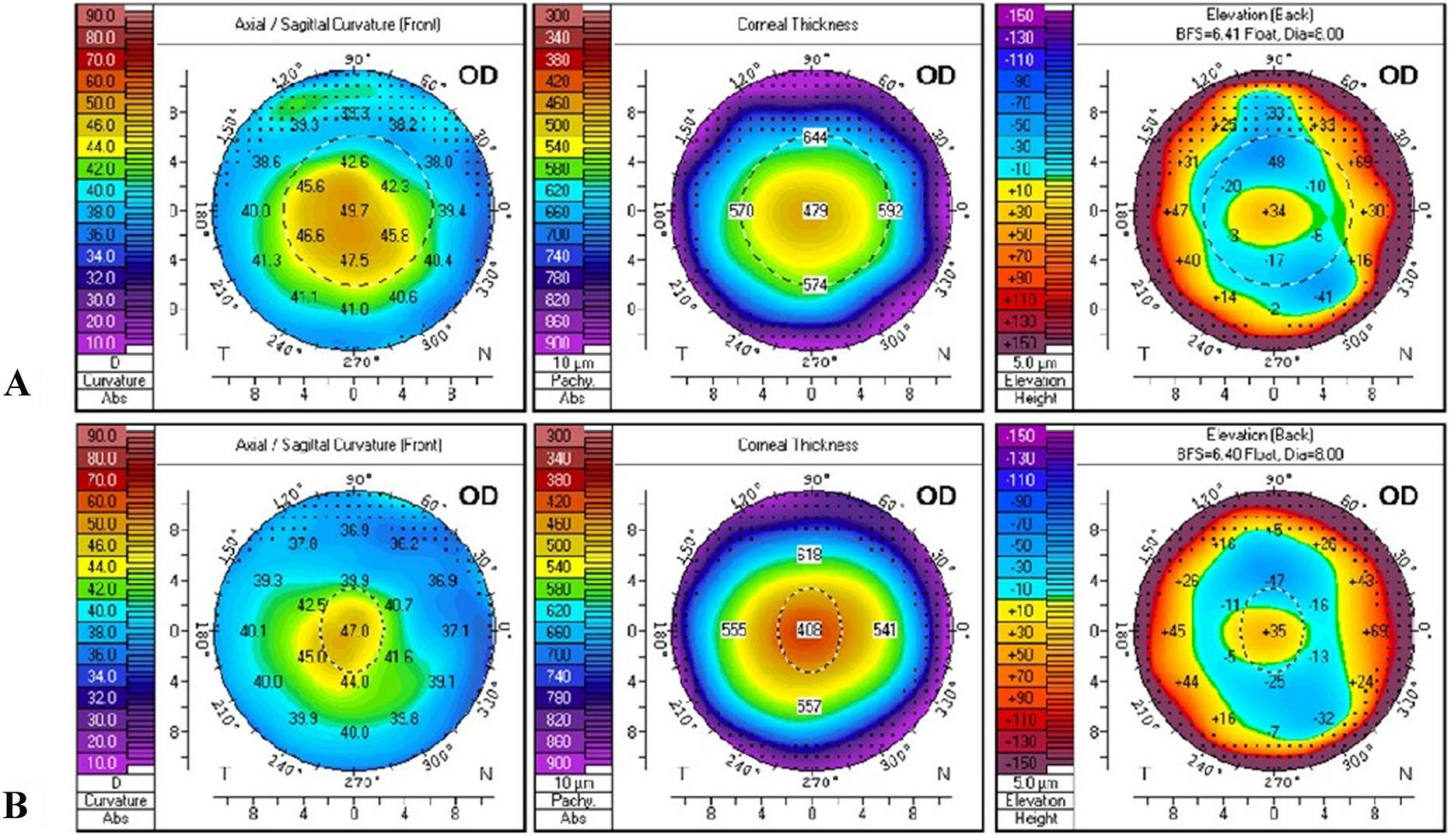
**A**: Preoperative, **B**: 6 months post PRK + CXL Scheimpflug tomography of stage 2 KC patient (correction -2 D sphere, -2 D cylinder, ablation depth 52 µ)

In this prospective study, three groups of patients were evaluated. ***PRK* + *CXL group*** included 64 eyes of 34 patients (19 males and 15 females; age: 19-40y) with stages 1–2 keratoconus, aged above 18 years old, which had undergone combined wavefront-optimized photorefractive keratectomy by WaveLight EX500 (Alcon Laboratories; Ft Worth, TX, USA) and corneal collagen crosslinking by Avedro KXL CXL system (Avedro, Waltham, Mass., USA). Stage 1 or 2 keratoconus was defined based on the Amsler-Krumeich classification (i.e., minimum corneal thickness > 400 microns μm, mean keratometry readings < 53.00 D with myopia and/or astigmatism not more than 8.00 D, and no corneal scarring) [8–10]. PRK aimed to correct up to 5 D of myopia and/or astigmatism only if the corneal thickness allowed with a postoperative residual corneal thickness of at least 400 µm. (Figs. 1 and 2).

**Fig. 3:**
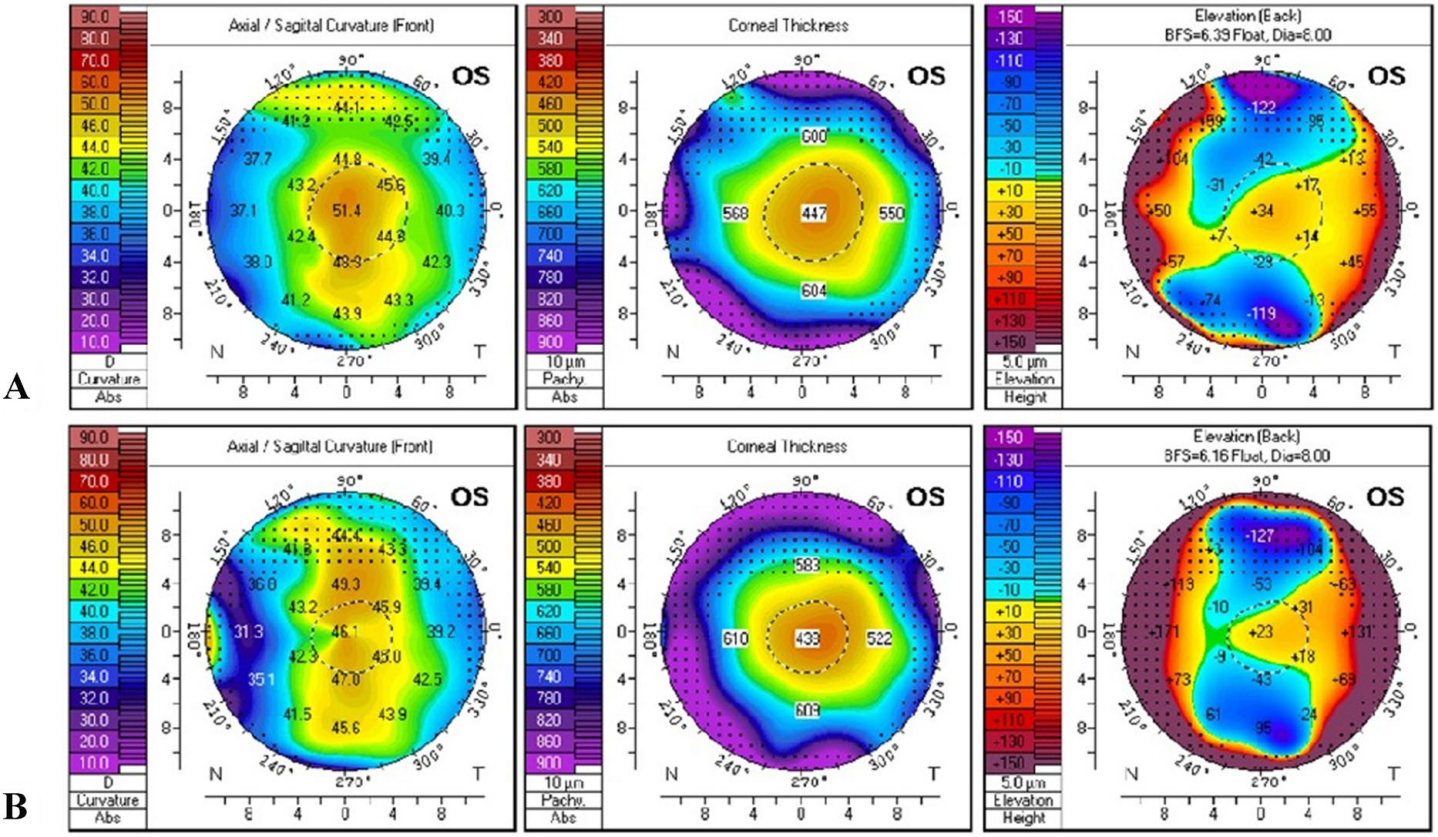
**A**: Preoperative, **B**: 6 months post CXL Scheimpflug tomography of stage 2 KC patient (unfit for PRK + CXL due to age, 15 years old)

***CXL group*** included 36 eyes of 23 patients (14 males and 9 females; age: 12-38y) with keratoconus, not filling the inclusion criteria for combined PRK and CXL, which had undergone corneal collagen cross-linking by Avedro KXL CXL system i.e. stages 3–4 keratoconus, age > 18 years old and corneal thickness not allowing a postoperative residual corneal thickness of 400 µm. (Fig. 3).

***PRK group*** included 110 eyes of 57 patients (23 males and 34 females; age: 18-44y) who had undergone wavefront-optimized photorefractive keratectomy by WaveLight EX500 (Alcon Laboratories; Ft Worth, TX, USA) for myopic refractive errors. Patients enrolled in the study had pre-operative pachymetry ≥ 460 μm and were required to retain an average post-operative residual corneal bed ≥ 380 μm.

Pregnant women, patients with corneal scarring, uncontrolled diabetes mellitus, collagen vascular diseases, severe xerophthalmia, corneal dystrophies, previous intraocular or refractive surgery, failure of follow-up, autoimmune disease, or history of herpetic keratitis were excluded from the three study groups.

### Clinical evaluation

Preoperative evaluation consisted of complete ophthalmic examinations, including autorefractometry (TOPCON RM-8900, Topcon Medical Systems, Tokyo, Japan), corneal topography assessment by Allegro Topolyzer-Vario (WaveLight, Erlagen, Germany), Scheimpflug tomography examination by Allegro Oculyzer II (WaveLight, Erlagen, Germany), and slitlamp examination of the anterior and posterior segments of the eye. IOP was measured by Goldman applanation tonometry (GAT) preoperatively and postoperatively at 3, 6 and 12 months follow-up visits.

### Surgical procedures

***PRK* + *CXL*** included PRK followed by accelerated epithelium-off CXL in the same setting.

***The first step:*** consisted of wavefront-optimized PRK by WaveLight EX500 (Alcon Laboratories; Ft Worth, TX, USA). 10 min preoperatively, topical anesthetic eye drops (Benox®, Eipico, Egypt) were instilled into the patient’s eye. Epithelial removal was performed using a hockey knife within an 8 mm zone, followed by PRK stromal ablation. A sponge soaked in 0.02% mitomycin C solution was applied onto the stromal surface for 20 s, then thoroughly washed with chilled saline solution, to minimize haze postoperatively.

***The second step*:** consisted of accelerated epithelium-off CXL using the Avedro KXL CXL system. Riboflavin (VibeX Rapid; Avedro Inc) was instilled onto the stromal surface for 10 min at 2-min intervals. Pulsed ultraviolet mode (1 s on and 1 s off) was applied for 8 min, delivering a total energy of 7.2 J/cm2 with a power of 30 mW/cm2.

***PRK group*** was subjected to the first step described, while the ***CXL group*** was subjected to the second step after epithelial removal by a hockey knife for an 8 mm zone.

Postoperatively in all studied groups, 1 drop of topical moxifloxacin 0.5% (Vigamox, Alcon) was instilled and a bandage contact lens (Focus Dailies, CIBA vision) was applied onto the cornea.

### Postoperative care and follow-up

All patients prescribed topical moxifloxacin 0.5% eye drops (Vigamox, Alcon) 4 times per day until complete corneal epithelial healing, fluorometholone 0.1% eye drops (Fluca, Jamjoom Pharma, Saudi Arabia) 4 times per day for 1 month then withdrawn gradually over 3 months, and lubricant eye drops (Systane, Alcon) 4 times per day for 1 month.

All eyes were examined twice during the first postoperative week, until complete corneal epithelium healing, after which the bandage contact lens was removed. Follow-up visits were weekly in the first postoperative month, and at 3, 6, and 12 months. During follow-up checks, the postoperative uncorrected distance visual acuity (UDVA), corrected distance visual acuity (CDVA), subjective refraction, spherical equivalent (SE), and IOP were recorded.

## Statistical analysis

Data were fed to the computer and analyzed using IBM SPSS software package version 20.0. **(**Armonk, NY: IBM Corp**)**. Categorical data were represented as numbers and percentages. The chi-square test was applied to compare the three studied groups. Quantitative data were expressed as range (minimum and maximum), mean, standard deviation and median. **Data were tested for normality by the Kolmogorov–Smirnov test.** For normally distributed quantitative variables, One way ANOVA test was used for comparing the three studied groups and followed by Post Hoc test (Tukey**)** for pairwise comparison. On the other hand, for not normally distributed quantitative variables, Mann Whitney test was used to compare two groups while Kruskal Wallis test was used to compare three groups and followed by Post Hoc test (Dunn's for multiple comparisons test) for pairwise comparison. Spearman coefficient was used to correlate between not normally distributed quantitative variables. Significance of the obtained results was judged at the 5% level. Sample size was calculated using Power Analysis and Sample Size Software (PASS 2020, NCSS, LLC. Kaysville, Utah, USA). Post-hoc power analysis is estimated to be 75%. A minimal total hypothesized sample size of 240 eyes (80 per group) was required to achieve 85% power; taking into consideration 95% confidence level and 80% power using Chi Square test.

## Results

The present study was conducted on 64 eyes of 34 patients (19 males and 15 females; age: 19-40y) with stages 1–2 keratoconus which had undergone combined wavefront-optimized photorefractive keratectomy and corneal collagen cross-linking PRK + CXL. Two other groups of patients were added as controls: the PRK group included 110 eyes of 57 patients (23 males and 34 females; age: 18-44y) which had undergone wavefront-optimized photorefractive keratectomy for myopic refractive errors, and the CXL group included 36 eyes of 23 patients (14 males and 9 females; age: 12–38y) with keratoconus, not filling the inclusion criteria for combined PRK and CXL, which had undergone corneal collagen cross-linking (Table 1).

**Table 1:** Comparison between the three studied groups according to demographic and topographic data

	PRK (n = 110)	CXL (n = 36)	PRK + CXL (n = 64)	Test of Sig	*p*
Age (years)					
Mean ± SD	28.5 ± 8.47	21.6 ± 8	28.2 ± 6.23	H = 23.282^*^	< 0.001^*^
Median (Min. – Max.)	26^a^ (18 – 44)	18^b^ (12 – 38)	27^a^ (19 – 40)		
Gender					
Male	46 (41.8%)	22 (61.1%)	35 (54.7%)	χ^2^ = 5.211	0.074
Female	64 (58.2%)	14 (38.9%)	29 (45.3%)		
K1 (D)					
Mean ± SD	42.9^c^ ± 2.25	46^a^ ± 4.24	44.2^b^ ± 1.90	F = 19.950^*^	< 0.001^*^
Median (Min. – Max.)	43.4 (34.7 – 47.2)	44.8 (35.4 – 54.8)	44 (40.3 – 48.5)		
K2 (D)					
Mean ± SD	44.6^c^ ± 2.37	49^a^ ± 5.67	46.4^b^ ± 2.16	F = 28.584^*^	< 0.001^*^
Median (Min. – Max.)	44.9 (36.8 – 48.9)	47.8 (37.9 – 61.4)	46.2 (41.5 – 51.5)		
Pachymetry apex (µ)					
Mean ± SD	509.5^a^ ± 30	456.6^c^ ± 29.5	479.6^b^ ± 34.8	F = 44.534^*^	< 0.001^*^
Median (Min. – Max.)	505 (440 – 582)	457 (404 – 512)	473.5 (402 – 573)		

**SD:** Standard deviation, **χ**^**2**^**:** Chi square test

**F:** F for One way ANOVA test. Pairwise comparison between each 2 groups was done using Post Hoc Test (Tukey)

**H:** H for Kruskal Wallis test. Pairwise comparison between each 2 groups was done using Post Hoc Test (Dunn's for multiple comparisons test)

**p:** p value for comparing between the three studied groups

^*^: Statistically significant at p ≤ 0.05

Means/Medians with any common letter ^(a−c)^ are not significant (or Means/Medians with totally different letters ^(a−c)^ are significant)

Preoperative IOP in both CXL (12.1 ± 2.53 mmHg) and PRK + CXL (13.2 ± 2.50 mmHg) groups was significantly lower than PRK group (15.8 ± 3.10 mmHg) (F = 30.505, *p* < 0.001). At 3 months postoperatively, IOP showed no statistically significant difference between the three studied groups (F = 1.821, *p* = 0.164). At 6 months postoperatively, IOP in the CXL group (14.6 ± 2.64 mmHg) was significantly higher than both PRK (13.4 ± 2.27 mmHg) and PRK + CXL (13.3 ± 2.62 mmHg) groups (F = 3.721, *p* = 0.026). At 12 months postoperatively, IOP in the CXL group (14.3 ± 2.69 mmHg) was significantly higher than the PRK group (13.2 ± 2.23 mmHg) and was higher than PRK + CXL group (13.3 ± 2.59 mmHg) although not statistically significant (F = 3.393, *p* = 0.035) (Tables 2 and 3).

**Table 2:** Comparison between PRK & PRK + CXL groups according to refractive surgical parameters

	PRK (n = 110)	PRK + CXL (n = 64)	U	p
Corrected spherical refractive error (D)				
Mean ± SD	-2.62 ± 2.01	-2.11 ± 1.42	3125.0	0.216
Median (Min. – Max.)	-2.25 (-8.25 – 0)	-2 (-5 – 0)		
Corrected cylinder (D)				
Mean ± SD	-1.57 ± 1.34	-1.66 ± 1.42	3402.0	0.711
Median (Min. – Max.)	-1.0 (-6 – 0)	-1.50 (-5 – 0)		
Corrected cylinder axis				
Mean ± SD	81.9 ± 72.2	72.1 ± 64	3281.50	0.456
Median (Min. – Max.)	74.5 (0 – 180)	50 (0 – 180)		
Corrected spherical equivalent (D)				
Mean ± SD	-3.43 ± 1.87	-2.98 ± 1.20	3308.00	0.507
Median (Min. – Max.)	-2.75 (-8.25 – -0.75)	-2.75 (-5.50 – -0.75)		
Ablation depth (µ)				
Mean ± SD	61.3 ± 25.7	51.1 ± 17.5	2795.50^*^	0.024^*^
Median (Min. – Max.)	58 (17 – 115)	52 (14 – 83)		
Percent of ablated tissue				
Mean ± SD	12.1 ± 5.10	10.7 ± 3.65	3057.50	0.149
Median (Min. – Max.)	11.2 (3.69 – 22.4)	10.9 (2.70 – 17.9)		

**SD:** Standard deviation**, U:** Mann Whitney test

**p:** p value for comparing between the two studied groups

*Statistically significant at p ≤ 0.05

**Table 3:** Comparison between the three studied groups according to IOP

IOP (mm Hg)	PRK (n = 110)	CXL (n = 36)	PRK + CXL (n = 64)	F	*p*
Preoperative					
Mean ± SD	15.8^a^ ± 3.10	12.1^b^ ± 2.53	13.2^b^ ± 2.50	30.505^*^	< 0.001^*^
Median (Min. – Max.)	16 (10 – 21)	11 (9 – 18)	13 (9 – 18)		
3 months postoperative					
Mean ± SD	13.1^a^ ± 2.19	13.9^a^ ± 2.66	13.2^a^ ± 2.56	1.821	0.164
Median (Min. – Max.)	13 (10 – 17)	13 (11 – 21)	13 (8 – 22)		
6 months postoperative					
Mean ± SD	13.4^b^ ± 2.27	14.6^a^ ± 2.64	13.3^b^ ± 2.62	3.721^*^	0.026^*^
Median (Min. – Max.)	13 (10 – 18)	14 (11 – 21)	13 (10 – 21)		
12 months postoperative					
Mean ± SD	13.2^b^ ± 2.23	14.3^a^ ± 2.69	13.3^ab^ ± 2.59	3.393^*^	0.035^*^
Median (Min. – Max.)	13 (10 – 17)	13 (12 – 21)	13 (10 – 21)		

**SD:** Standard deviation

**F:** F for One way ANOVA test. Pairwise comparison between each 2 groups was done using Post Hoc Test (Tukey)

**p:** p value for comparing between the three studied groups

*Statistically significant at p ≤ 0.05

Means with any common letter ^(a−c)^ are not significant (or means with totally different letters ^(a−c)^ are significant)

**Fig. 4:**
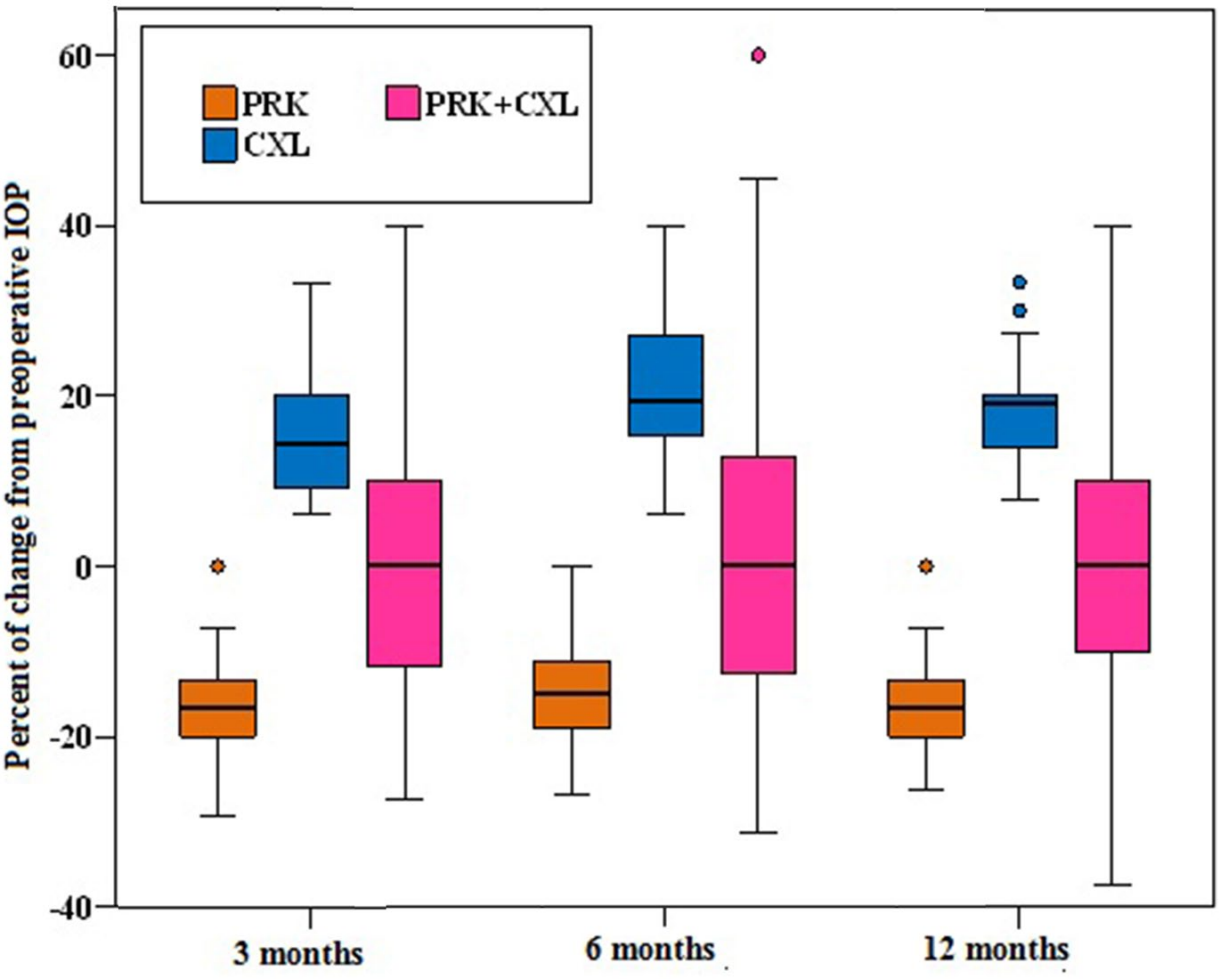
Comparison between the three studied groups according to percent of change from preoperative IOP

Regarding the percent of change from preoperative IOP, a statistically significant difference between the three studied groups was detected at 3, 6 and 12 months postoperatively (H = 117.459, 109.303, 122.694 respectively, *p* < 0.001). The median percent of change from preoperative IOP in the PRK group was −16.7%, −15.0%, and −16.7%, in the CXL group was + 14.3%, + 19.4%, and + 19.1%, while in PRK + CXL group was 0% at 3, 6 and 12 months postoperatively. (Table 4) (Fig. 4).

**Table 4:** Comparison between the three studied groups according to percent of change from preoperative IOP

Percent of change from preoperative IOP	PRK (n = 110)	CXL (n = 36)	PRK + CXL (n = 64)	H	*p*
3 months postoperative					
Mean ± SD	-16.6 ± 6.4	15.3 ± 7.1	1.4 ± 15.3	117.459^*^	< 0.001^*^
Median (Min. – Max.)	-16.7^c^ (-29.4 – 0)	14.3^b^ (6.3 – 33.3)	0^a^ (-27.3 – 40)		
6 months postoperative					
Mean ± SD	-14.5 ± 6.1	21 ± 8.6	2.8 ± 17.8	109.303^*^	< 0.001^*^
Median (Min. – Max.)	-15^c^ (-26.7 – 0)	19.4^a^ (6.3 – 40)	0^b^ (-31.3 – 60)		
12 months postoperative					
Mean ± SD	-16 ± 5.6	18.7 ± 6.4	2 ± 16.3	122.694^*^	< 0.001^*^
Median (Min. – Max.)	-16.7^c^ (-26.3 – 0)	19.1^a^ (7.7 – 33.3)	0^b^ (-37.5 – 40)		

**SD:** Standard deviation

**H:** H for Kruskal Wallis test. Pairwise comparison between each 2 groups was done using Post Hoc Test (Dunn's for multiple comparisons test)

**p:** *p* value for comparing between the three studied groups

^*^: Statistically significant at *p* ≤ 0.05

Medians with any common letter ^(a−c)^ are not significant (or medians with totally different letters ^(a−c)^ are significant)

PRK group showed a significant reduction in IOP at 3, 6, and 12 months postoperatively by 16.6 ± 6.4%, 14.5 ± 6.1%, and 16 ± 5.6% respectively. This reduction correlated significantly with the corrected spherical refractive error (r_s_ 0.226, 0.199, 0.212, p 0.017, 0.037, 0.026 at 3, 6 and 12 months postoperatively respectively), the corrected cylindrical refractive error (r_s_ 0.221, 0.249, p 0.020, 0.009 at 3, and 6 months postoperatively respectively), ablation depth (r_s_ −0.264, −0.242, −0.218, p 0.005, 0.011, 0.022 at 3, 6 and 12 months postoperatively respectively), and percent of ablated tissue (ablation depth / pachymetry apex) (r_s_ −0.274, −0.239, −0.212, p 0.004, 0.012, 0.026 at 3, 6 and 12 months postoperatively respectively). The most statistically significant factors affecting percent of IOP reduction were ablation depth and percent of ablated tissue. (Tables 4 and 5).

**Table 5:** Correlation between Percent of change in IOP with different parameters in PRK group

		Percent of change in IOP at		
		3 months	6 months	12 months
K1 (D)	r_s_	-0.007	0.097	-0.034
	p	0.943	0.313	0.722
K2 (D)	r_s_	-0.098	0.009	-0.119
	p	0.307	0.923	0.216
Pachymetry apex (µ)	r_s_	0.079	0.009	-0.096
	*p*	0.411	0.930	0.316
Spherical refractive error (D)	r_s_	0.226^*^	0.199^*^	0.212^*^
	*p*	0.017^*^	0.037^*^	0.026^*^
Cylinder (D)	r_s_	0.221^*^	0.249^*^	0.156
	*p*	0.020^*^	0.009^*^	0.104
Spherical equivalent (D)	r_s_	0.100	0.068	0.107
	*p*	0.296	0.479	0.266
Ablation depth (µ)	r_s_	-0.264^*^	-0.242^*^	-0.218^*^
	*p*	0.005^*^	0.011^*^	0.022^*^
Percent of ablated tissue	r_s_	-0.274^*^	-0.239^*^	-0.212^*^
	*p*	0.004^*^	0.012^*^	0.026^*^

**r**_**s**_**:** Spearman coefficient

*Statistically significant at *p* ≤ 0.05

CXL group showed a significant increase in IOP at 3, 6, and 12 months postoperatively by 15.3 ± 7.1%, 21 ± 8.6%, and 18.7 ± 6.4% respectively. This increase correlated significantly with preoperative K1 (r_s_ 0.388, 0.434, p 0.020, 0.008 at 3, and 6 months postoperatively respectively), preoperative K2 (r_s_ 0.336, 0.330, p 0.045, 0.049 at 3, and 6 months postoperatively respectively), and preoperative pachymetric apex (r_s_ −0.554, −0.637, −0.498, *p* < 0.001, < 0.001, 0.002 at 3, 6 and 12 months postoperatively respectively). The most statistically significant factor affecting the percent of IOP elevation was the preoperative pachymetric apex (Tables 4 and 6).

**Table 6:** Correlation between percent of change in IOP with different parameters in CXL group

		Percent of change in IOP at		
		3 months	6 months	12 months
K1 (D)	r_s_	0.388^*^	0.434^*^	0.298
	*p*	0.020^*^	0.008^*^	0.077
K2 (D)	r_s_	0.336^*^	0.330^*^	0.234
	*p*	0.045^*^	0.049^*^	0.169
Pachymetry apex (µ)	r_s_	-0.554^*^	-0.637^*^	-0.498^*^
	*p*	< 0.001^*^	< 0.001^*^	0.002^*^

**r**_**s**_**:** Spearman coefficient

*Statistically significant at *p* ≤ 0.05

PRK + CXL group showed minor change in IOP at 3, 6, and 12 months postoperatively by 1.4 ± 15.3%, 2.8 ± 17.8%, and 2 ± 16.3% respectively. This change correlated significantly with the corrected cylindrical refractive error (r_s_ 0.287, p 0.021 at 3 months postoperatively), preoperative pachymetric apex (r_s_ 0.268, p 0.032 at 3 months postoperatively), ablation depth (r_s_ −0.317, p 0.011 at 3 months postoperatively), and percent of ablated tissue (r_s_ −0.388, −0.291, p 0.002, 0.020 at 3, and 6 months postoperatively respectively) (Tables 4 and 7).

**Table 7:** Correlation between percent of change in IOP with different parameters in PRK + CXL group

		Percent of change in IOP at		
		3 months	6 months	12 months
K1 (D)	r_s_	0.119	0.190	0.148
	p	0.350	0.132	0.244
K2 (D)	r_s_	-0.105	0.020	0.009
	p	0.410	0.875	0.942
Pachymetry apex (µ)	r_s_	0.268^*^	0.242	0.161
	p	0.032^*^	0.054	0.205
Spherical refractive error (D)	r_s_	0.172	0.097	0.065
	p	0.174	0.444	0.612
Cylinder (D)	r_s_	0.287^*^	0.227	0.191
	p	0.021^*^	0.072	0.130
Spherical equivalent (D)	r_s_	-0.042	-0.044	-0.054
	p	0.741	0.730	0.670
Ablation depth (µ)	r_s_	-0.317^*^	-0.220	-0.161
	p	0.011^*^	0.081	0.205
Percent of ablated tissue	r_s_	-0.388^*^	-0.291^*^	-0.212
	p	0.002^*^	0.020^*^	0.092

**r**_**s**_**: Spearman coefficient**

^*^: Statistically significant at *p* ≤ 0.05

## Discussion

Keratoconus is a bilateral progressive, usually asymmetric, non-inflammatory corneal ectatic disorder, characterized by increased corneal curvature and decreased corneal thickness. These changes in corneal parameters not only reduce image quality and visual acuity, but also affect intraocular pressure.

In the present study, preoperative GAT measured IOP in KC eyes, including both CXL (12.1 ± 2.53 mmHg) and PRK + CXL (13.2 ± 2.50 mmHg) groups, was significantly lower than non-KC eyes i.e. PRK group (15.8 ± 3.10 mmHg) (F = 30.505, *p* < 0.001). These results are in agreement with Ortiz et al., who reported a significant difference in GAT-measured IOP between normal (16.3 ± 3.5 mmHg) and KC eyes (11.4 ± 2.9 mmHg). Similarly, Firat et al., reported statistically significant lower GAT measured IOP in KC eyes [10 (4–16) mmHg] compared to normal eyes [14 (9–17) mmHg] (*p* < 0.0001) [11, 12].

The IOP values in KC eyes reported in the present study coincide with values reported by Kymionis et al. (9.95 ± 3.01 mmHg) and Sanja et al. [12 (10,62–15,25) mmHg] [6, 13]. Lower GAT measured IOP in KC eyes could be attributed to reduced corneal stiffness, corneal stromal thinning, reduced number of collagen stromal lamellae, and decreased keratocyte density which reduces stromal actin production and destabilizes corneal stromal cytoskeleton [14, 15].

Cross-linking involves the creation of covalent bonds between polymer molecules, increasing chemical strength. The approach of KC treatment by cross-linking was first introduced by a research team at Dresden Technical University (Germany) in the 1990s [16, 17].

In the present study, the CXL group showed a statistically significant increase in IOP at 3, 6 and 12 months postoperatively (13.9 ± 2.66, 14.6 ± 2.64, and 14.3 ± 2.69 mm Hg respectively). This increase in IOP goes in agreement with studies by Kymionis et al. (11.40 ± 2.89, and 11.35 ± 3.38 mm Hg at 6 and 12 months postoperatively respectively, both *p* < 0.01), and Sanja et al. [13,5 (11–16), 14 (11–16), and 15 (10,37–17,25) mmHg at 3, 6 and 12 months postoperatively respectively, *p* = 0,010 at 12 months postoperatively compared to preoperative IOP] [6, 13]. Similarly, Kaufman et al. reported a statistically significant increase in Tonopen (Reichert. Depew, NY USA) measured IOP at 1 (17.1 ± 2.7 mm Hg), 3 (15.8 ± 2.3 mmHg), and 6 months (16.8 ± 1.8 mmHg) postoperatively [18].

The increase in IOP reported after CXL can be explained by an increase in corneal stiffness and mechanical rigidity owing to inter- and intra-fibrillar corneal collagen cross-links, together with an increase in collagen fiber diameter of about 10% after CXL, particularly in the anterior corneal stroma [6, 13, 19].

On the other hand, Waheed et al. reported a statistically significant decrease in GAT measured IOP on the same day after CXL (*P* = 0.001), with no statistically significant change in IOP on the 2nd day, at 1 month and 3 months postoperatively. These results could be attributed to the fact that Waheed et al. applied epithelium-on (epi-on) CXL technique, raising further concerns regarding the efficacy of epi-on CXL when compared to standard epi-off CXL [20–22].

In the present study, the increase in IOP after CXL correlated significantly with preoperative K1 (r_s_ 0.388, 0.434, p 0.020, 0.008 at 3, and 6 months postoperatively respectively), preoperative K2 (r_s_ 0.336, 0.330, p 0.045, 0.049 at 3, and 6 months postoperatively respectively), and preoperative pachymetric apex (r_s_ −0.554, −0.637, −0.498, *p* < 0.001, < 0.001, 0.002 at 3, 6 and 12 months postoperatively respectively). The most statistically significant factor affecting the percent of IOP elevation was the preoperative pachymetric apex. On the other hand, Kymionis et al. reported that the increase in IOP at 6 and 12 months post-CXL was not correlated with preoperative pachymetry (*p* = 0.113, 0.522 respectively), or preoperative K readings (*P* = 0.061, 0.324, respectively) [6].

In the present study, the PRK group showed a significant reduction in IOP at 3, 6, and 12 months postoperatively by 16.6 ± 6.4%, 14.5 ± 6.1%, and 16 ± 5.6% respectively. This reduction correlated significantly with the corrected spherical refractive error (r_s_ 0.226, 0.199, 0.212, p 0.017, 0.037, 0.026 at 3, 6 and 12 months postoperatively respectively), the corrected cylindrical refractive error (r_s_ 0.221, 0.249, p 0.020, 0.009 at 3, and 6 months postoperatively respectively), ablation depth (r_s_ −0.264, −0.242, −0.218, p 0.005, 0.011, 0.022 at 3, 6 and 12 months postoperatively respectively), and percent of ablated tissue (ablation depth /pachymetry apex) (r_s_ −0.274, −0.239, −0.212, p 0.004, 0.012, 0.026 at 3, 6 and 12 months postoperatively respectively). The most statistically significant factors affecting the percent of IOP reduction were ablation depth and percent of ablated tissue, suggesting decreased corneal thickness as the leading etiology for IOP reduction.

These results go in agreement with Schallhorn et al., who reported an IOP reduction after myopic PRK estimated at 0.021 mmHg/µm ablated tissue, and 0.4 mmHg / D of corrected myopia (95% CI) [7]. Similarly, Chou et al. reported a statistically significant correlation between IOP reduction after PRK with corrected spherical equivalent (*p* = 0.254) and ablation depth (*p* = 0.278) [23].

Based on earlier studies, keratoconic eyes who underwent sequential topography-guided PRK six months to one year following CXL experienced a significant improvement in their functional vision, however this sequential treatment protocol has some drawbacks, namely a higher incidence of post-PRK haze, the difference in the ablation rate between normal and cross- linked corneas, and most importantly, PRK ablation of CXL stiffened corneal stoma jeopardizing the benefits of CXL [24]. Later, Kanellopoulos introduced another technique called CXL-Plus, which involves same-day simultaneous topography-guided PRK followed by CXL (the Athens protocol). The main advantage of combined PRK and CXL over sequential PRK after CXL is the non-affection of the cross-linked corneal stroma by excimer laser ablation [2, 25].

The Athens protocol involved the use of topography-guided PRK for correction of up to 1 D of the myopic component and up to 2.5 D of the astigmatic component of keratoconus, by ablating a maximum of 50 µm from the anterior corneal stroma in advanced keratoconus to reduce anterior corneal surface irregularities followed by CXL [2, 25]. In contrast, in our study, PRK + CXL group involved stages 1–2 keratoconus (K1 44.2 ± 1.90 D, K2 46.4 ± 2.16 D, and pachymetric apex 479.6 ± 34.8 µm) which had undergone combined wavefront optimized PRK followed by CXL. In our study, PRK corrected up to 5 D of myopia (−2.11 ± 1.42 D) and/or astigmatism (−1.66 ± 1.42 D) provided that a minimum corneal thickness of 400 µm at the thinnest location remained postoperatively. The ablation depth in our studied PRK + CXL group ranged from 14 to 83 µm (51.1 ± 17.5 µm).

Iqbal et al. applied standard non-topography guided PRK (VISX S4; Maloney Vision Institute, Los Angeles, CA) followed by epithelium off CXL in stage 1 KC (K1 44.75 ± 1.18, K2 48.28 ± 0.66, and pachymetric apex 473 ± 29 µm), correcting 3.65 ± 1.72 D of myopia and 1.83 ± 0.69 D of astigmatism and reported significant visual and refractive improvement over 18 months of follow-up [26].

De Rosa et al. applied topography-guided PRK (WaveLight ALLEGRETTO WAVE® Eye-Q, ALCON, Fort Worth, TX, USA), followed by standard CXL in stages 1–2 KC (K1 45.18 ± 2.17, and K2 48.79 ± 3.22), correcting 40% of the myopic refractive error (3.65 ± 2.01 D) and 70% of the cylinder (3.63 ± 1.67 D) respecting a maximal ablation depth of 50 µm, reporting safety and efficacy with 24-months refractive stability [27].

Al Amri reported significant visual and refractive improvement over 5 years of follow-up after combined non-topography guided PRK (Quest excimer laser platform, NIDEK Co. Ltd., Japan), followed by CXL in stages 1–2 KC (K1 43.82 ± 2.8, K2 48.6 ± 3.1, and pachymetric apex 496.1 ± 12.97 µm), correcting 1.62 ± 1.23 D of myopia and 1.73 ± 0.86 D of astigmatism [3].

Iqbal et al. compared standard 30 min epithelium‐off CXL (group A) versus PRK combined with accelerated epithelium‐off cross‐linking (AXL) (group B) for progressive keratoconus management, and reported close results at the 24th follow‐up month. Postoperative spherical equivalent reduction at the end of the 24th postoperative month was 2.15 ± 0.67 D (*p* < 0. 001) in group A, and 2.23 ± 0.49 D (*p* value < 0. 001) in group B, however, standard CXL seemed to be more powerful than AXL in its long‐term effect [28].

In the present study, the median percent of change from preoperative IOP in PRK + CXL group was 0% at 3, 6 and 12 months postoperatively, with mean IOP values preoperatively and at 3, 6 and 12 months postoperatively 13.2 ± 2.50, 13.2 ± 2.56, 13.3 ± 2.62, and 13.3 ± 2.59 mmHg respectively. This could be explained by the fact that IOP reduction induced by PRK is compensated by increased corneal stiffness and mechanical rigidity induced by CXL. One of the limitations of the present study is the inclusion of both eyes of the same subject, which might be a source of statistical bias. Another limitation of our study is that Post-hoc power analysis is estimated to be 75%. A minimal total hypothesized sample size of 240 eyes (80 per group) was required to achieve 85% power; taking into consideration 95% confidence level and 80% power using Chi Square test.

To our knowledge, this is the first published study investigating the effect of combined PRK and CXL on IOP in keratoconus.

## Data Availability

Datasets are available in additional supporting files.
